# Genetics of hearing loss in Africans: use of next generation sequencing is the best way forward

**DOI:** 10.11604/pamj.2015.20.383.5230

**Published:** 2015-04-17

**Authors:** Kamogelo Lebeko, Jason Bosch, Jean Jacques Nzeale Noubiap, Collet Dandara, Ambroise Wonkam

**Affiliations:** 1 Division of Human Genetics, Faculty of Health Sciences, University of Cape Town, Cape Town, South Africa; 2 Faculty of Medicine and Biomedical Sciences, University of Yaoundé I, Yaoundé, Cameroon; 3 Institute for Infectious Disease and Molecular Medicine (IDM), Faculty of Health Sciences, University of Cape Town, Cape Town, South Africa

**Keywords:** ARNSHL, Sub-Saharan Africa, next generation sequencing, whole exome sequencing, targeted exome sequencing, hearing loss, OTOScope^®^

## Abstract

Hearing loss is the most common communication disorder affecting about 1-7/1000 births worldwide. The most affected areas are developing countries due toextensively poor health care systems. Environmental causes contribute to 50-70% of cases, specifically meningitis in sub-Saharan Africa. The other 30-50% is attributed to genetic factors. Nonsyndromic hearing loss is the most common form of hearing loss accounting for up to 70% of cases. The most common mode of inheritance is autosomal recessive. The most prevalent mutations associated with autosomal recessive nonsyndromic hearing loss (ARNSHL) are found within connexin genes such as GJB2, mostly in people of European and Asian origin. For example, the c.35delG mutation ofGJB2 is found in 70% of ARNSHL patients of European descentand is rare in populations of otherethnicities. Other GJB2 mutations have been reported in various populations. The second most common mutations are found in theconnexin gene, GJB6, also with a high prevalencein patients of European descent. To date more than 60 genes have been associated with ARNSHL. We previously showed that mutations in GJB2, GJB6 and GJA1 are not significant causes of ARNSHL inpatients from African descents, i.e. Cameroonians and South AfricansIn order to resolve ARNSHL amongst sub-Saharan African patients, additional genes would need to be explored. Currently at least 60 genes are thought to play a role in ARNSHL thus the current approach using Sanger sequencing would not be appropriate as it would be expensive and time consuming. Next Generation sequencing (NGS) provides the best alternative approach. In this review, we reported on the success of using NGSas observed in various populations and advocate for the use of NGS to resolve cases of ARNSHL in sub-Saharan African populations.

## Introduction

Hearing loss is defined as disabling when the loss of hearing is greater than 40dB in the better hearing ear [1]. It is listed as the 12^th^ most common contributor to disease burden globally [2]. Hearing loss is the number one communication disorder in the world affecting about 360 million individuals, 32 million of whom are children under the age of 15 [1]. It affects about 1 in 1000 live births in developed countries and about 3 in 1000 births in developing countries [1]. Prevalence of hearing loss is highest in South Asia and sub-Saharan Africa [3] which are part of thelow income regions or the developing world [1]. This is attributed to poor health care systems where complications at birth as well as infections could result in loss of hearing in the new-born [4]. In the Philippines, a prevalence rate of 6 per 1000 was reported which is comparable to the 7 per 1000 and 6 per 1000, in Nigeria and South Africa, respectively [5].

There are various classifications used to describe the clinical manifestation of hearing loss. The American Speech-Language-Hearing Association (ASHA) classifies hearing loss according to the following characteristics [6]: **pathophysiology** refers to the component of the hearing system which is non-functional. This can be any of the outer, middle or inner ear components. Conductive hearing loss refers to the non-functionality of the outer or middle ear. Sensorineural refers to hearing loss due to non-functionality of the inner ear. When both the inner and outer or middle ear is not functioning, it is referred to as mixed hearing loss; **severity** is determined by measuring the threshold needed for sound to be perceived. It is determined by generating an audiogram. The higher the threshold where sound is perceived, the more severe the loss of hearing; onset is either pre-lingual, which is before the development of speech as seen in congenital cases, or post-lingual. If the hearing loss in both ears then it is referred to as bilateral and unilateral if only one ear is affected.

We reviewed the available studies on the aetiology and genetics of hearing loss in Africa, including our previous publications on this topic that have shown the non-implication in Africansof the genes most commonly associated with ARNSHL among patients of European and Asian descent: GJB2 and GJB6. In this article, we review the success of using NGS as done so by other research groups in various populations and advocate for the use of NGS to resolve cases of ARNSHL in sub-Saharan African populations. This will provide rapid results which will contribute towards building a genetic profile on ARNSHL amongst sub-Saharan Africans.

## Methods

The present article is a systematic review of most recent publications of hearing loss with focus on relevant articles on aetiology of hearing loss in sub-Saharan Africa and those that involved NGS to study the causes of hearing loss. The key word used was hearing loss, aetiology, genetics, Africa. We used the following search engines: PubMed^®^ and Google Scholar^®^ (August 2014). Only publications in English, were retrieved and included in the manuscript.

## Current status of knowledge

### Aetiology of hearing loss: environmental causes

The causes of hearing loss can either be genetic or environmental. In developing communities, the environment contributes significantly more to the incidence of congenital hearing loss than in the developed world [2]. This is attributed to limited access to healthcare systems that are not always adequately equipped to assist and monitor pregnancy and birth. Malnutrition during pregnancy may lead to low birth weight which may result in complications which could lead to hearing loss [2]. The lack of gestational vitamin A has been suggested to contribute to hearing loss development in developing countries [7]. Other environmental factors which contribute to cases of non-genetic congenital hearing loss are trauma which is most common in areas where mothers give birth unassisted or by poorly trained staff and infection of viral diseases which affect the unborn child such as infection with Cytomegalovirus [8].

Specifically in Africa, an infection which contributes to cases of hearing loss in infants and young children is that of bacterial meningitis. In a study in Kenya [9] and many other African countries [10–14], it was observed that there was a high prevalence of sensorineural hearing loss amongst children treated for bacterial meningitis (Table 1). Children living in developing countries who fail to get vaccinated are at a greater risk of developing hearing loss sequel to bacterial meningitis infection [15].

**Table 1:** Comparison of aetiological studies on hearing loss in sub-Saharan Africa

Country	Gambia	Nigeria	Sierra Leone	Ghana	Cameroon
Year of Study	1985 [10]	1982 [11]	1998 [12]	1988 [13]	2013 [14]
Number of Patients	259	298	354	105	582
Hereditary	8.1%	13.1%	-	-	14.8%
Meningitis	30%	11%	23.9%	8.5%	34.4%
Measles	1.9%	13%	4.1%	30%	4.3%
Rubella	1.5%	2%	-	3.5%	0.5%
Mumps	-	3%	16.7%	3.5%	2.1%
Ototoxicity	-	9%	20.8%	-	6%

There has been some investigation into genes that are thought to be candidates for differential susceptibility of certain individuals to noise-induced hearing loss (NIHL) while others seem unaffected [16]. A more complex incident of hearing loss is age related hearing loss (ARHL). The elderly account for the highest proportion of hearing-impaired people in the world. Within the age group of 65 and older, one in three individuals will experience some degree of hearing loss[1]. The complexity of ARHL is due to the interaction between environment factors, clinical history i.e. medication, social behaviour e.g. smoking, drinking, and genetic factors [17]. Much like with NIHL, there haven't been many genes isolated as candidate genes for susceptibility to ARHL. In cases of congenital hearing loss, the absence of known environmental influence leads to the assumption that a genetic factor is the cause of hearing loss

### Aetiology of hearing loss: genetics of congenital hearing loss

#### The genes

There is high variability in the genes causing hearing loss as well as their causative mutations. To date, there are over 60 genes implicated in cases of hearing loss [18].

#### Clinical features and heredity

**Figure 1:**
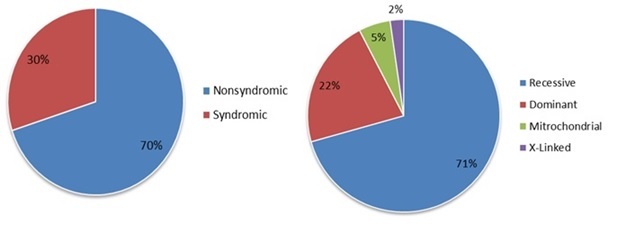
Proportion of syndromic, nonsyndromicand mode of inheritance of hearing loss. (adapted from [21])

The manifestation of the hearing loss may be syndromic whereby there are other clinical features associated with the loss of hearing. On the other hand, it might be nonsyndromic whereby hearing loss is the only observed symptom. Nonsyndromic hearing loss accounts for up to 75% of cases of putative genetic origin [19]. Syndromic and nonsyndromic cases of hearing loss can be caused by mutations which act in a dominant or recessive manner. They can be autosomal or X-linked and some have been identified on mitochondrial DNA. Fifty percent of the cases are genetic with autosomal recessive non-syndromic hearing loss (ARNSHL) being the most common [20]. Figure 1 illustrates that the most common mode of inheritance in nonsyndromic hearing loss.

### Syndromic hearing loss

#### Usher syndrome

This syndrome is characterised by hearing loss and retinitispigmentosa. It accounts for 50% of cases of combined deafness and blindness worldwide. It has three clinical subtypes classified according to the severity of the hearing loss [21]. The most prevalent mode of inheritance for Usher syndrome is autosomal recessive [22]. Up to 11 genes to date have been identified with causative mutations which lead to the development of profound congenital hearing loss or progressive hearing loss. Genes involved in Usher syndromes include *MYO7A, CDH23*, and *USH1C* to name a few. *MYO7A* has been implicated in various populations as harbouring causative mutations for ARNSHL [23, 24]. There is a scarcity of epidemiological data in Sub-Saharan African populations with only one case reported in Cameroon in 2013 [14].

#### Pendred syndrome

This is an autosomal recessive syndrome with symptoms such as sensorineural hearing loss andpartially defective iodine organification, a process of adding iodine to thyroglobin for the production of thyroid hormone[25]. It accounts for up to 10% of all cases of genetic hearing loss [26]. Mutations in SLC26A, which encodes for Pendrin, have been associated with Pendred syndrome. It is a homogeneous syndrome as all patients with biallelicmutations in SLC26A have Pendred syndrome. Pendrin functions as an anion exchanger and is expressed in the kidney, thyroid and inner ear. Loss of hearing is caused by increased calcium concentrations in the endolymph disrupting signalling transduction [26].

### Keratitis-Ichthyosis-Deafness syndrome

**Figure 2:**
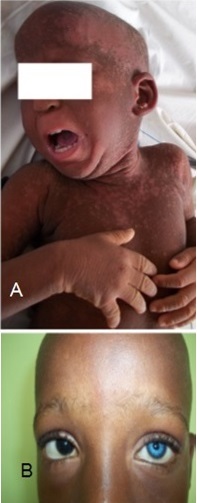
Clinical phenotype of selected syndromes: (a) cameroonian patient presenting with erythrokeratoderma, a symptom of KID; (B) *Heterochromia iridis* in a cameroonian patient with Waardenburg syndrome

This is a congenital disorder characterised by profound hearing loss, hyperkeratosis (thickening of the skin) and erythrokeratoderma (scaly skin) (Figure 2 (A)). It affects the eye as well: Keratitis in the name refers to the inflammation of the cornea. The major genes implicated in KID are connexin genes *GJB2* and *GJB6* [27]. The most prevalent mutation associated with KID is p.Asp50Asn in *GJB2* [28]. A recent publication has also reported two cases in sub-Saharan Africa of sporadic origin carrying the above mentioned mutation [29]. This indicates that the mutation is not population specific.

### Waardenburg syndrome

It is described as an auditory-pigmentory disorder which affects the iris, hair and skin's pigmentory deposits (Figure 2 (B)) [30]. Hearing loss associated with Waardenburg syndrome is usually of congenital sensorineural type with about 40% of cases displaying progressive hearing loss. It is inherited in an autosomal dominant manner [31]. Major genes in Waardenburg syndrome are PAX3 and SOX10 [30]. Waardenburg has been noted as the most frequent hearing loss syndrome amongst sub-Saharan African patients [32].

### Oculo-auriculo-vertebral (OAV) spectrum (Goldenhar Syndrome)

This is a rare congenital disorder that affects the development of the ear, nose and soft palate [33]. There are other anomalies which might present within the spectrum. The cause is largely unknown though it is thought to have a genetic component [34]. Given the rarity of the syndrome and its genetic heterogeneity, it is not unexpected that there is little data freely available on cases of Goldenhar Syndrome amongst sub-Saharan patients. Following aPubMed search for “Goldenhar Syndrome” “Africa”, three papers appeared with only one being freely accessible. It was a case report in 1998 of a 15-month old from South Africa with ameloblastic fibroma associated with OAV [35].

### Nonsyndromic hearing loss

#### Connexin genes and ARNSHL in the global population

Autosomal recessive nonsyndromic hearing loss (ARNSHL) is the most common type of hearing loss [36]. The most common mutations associated with ARNSHL are found within connexin genes. They have been implicated amongst European populations of Caucasian descent, as well as in Mediterranean populations such as Spain, Italy, France, and also among Arab populations in Middle East and North Africa. Mutations in GJB2, which encodes for connexin 26, are the most common cause of hearing loss amongst this population. The most common GJB2 mutation is c.35delG which is seen in up to 70% of cases [37]. The second most common gene associated with hearing loss is GJB6 which encodes for connexin 30 [38]. Outside of the Caucasian European population, the implication of c.35delG in hearing loss has been rare leading to the hypothesis that this is a founder mutation amongst populations of Caucasian descent [39]. Mutations uncovered in these two connexin genes have allowed for a rapid diagnostic approach within this population. Up to 70% of the cases of hearing loss are resolvedby screening for the most common mutations followed by screening the entire gene.

**Figure 3:**
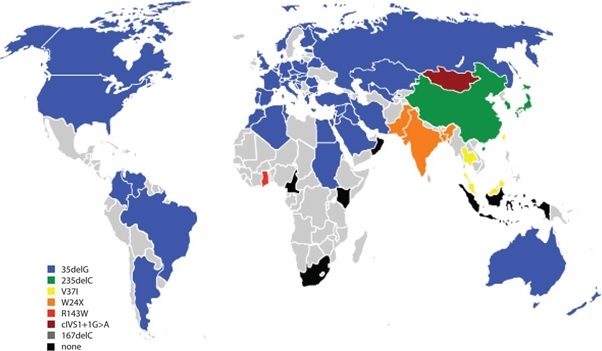
The most common *GJB2* mutations found amongst various World populations. Countries shown in grey do not have published data on GJB2 mutations, the majority in sub-Saharan Africa (adapted from [40])

Screening of GJB2 has been conducted in other populations [40] such as in Asian populations where 35delG has not been detected, which further corroborates the founder effect hypothesis. Instead, a different founder mutation for this population group was identified as 235delC [41]. Amongst Mediterranean populations and Ashkenazi Jews, 167delT is the most prevalent GJB2 mutation, also hypothesized to be due to a founder mutation [36, 42]. Figure 3 shows the most common GJB2 mutations within their respective populations.

#### Connexin genes and ARNSHL in africans

In Sub-Saharan Africa, *GJB2* has been investigated in various countries. It was only implicated in Ghana with p.R43W being the most common mutation for this population [43]. Screening in Cameroon [44], South Africa [45] and Kenya [46] did not reveal any significant *GJB2* mutations. *Wonkamet al* reported two cases of KID syndrome due to mutations in *GJB2* and illustrated that these cases have the most common mutation in the global population [29]. In addition Bosch et al extracted data for the 1000 Genome project and compared it to that of the GJB2 sequences from both Cameroon and South Africa and showed that there was a low level of variance[47]. Together, with the founder effect of the GJB2 mutation reported in Caucasian population [37, 48, 49], these data indicated that the African DNA are not “immune” of GJB2 mutations, which seem to havein Eurasian populations after their migration out of Africa, and spread with population migrations.

*GJB6* has also been investigated in other populations and its variants are yet to be identified outside of the European Caucasian population [45, 50–55]. We recently showed that major *GJB6* mutations are not of significance in sub-Saharan African populations and that there is little evidence to suggest that rare causative mutations might be harboured by this population group [56]. We also screened the coding region of *GJB6* to try and uncover any rare point mutations [56]. The results suggested that *GJB6* genetic variation is not a significant factor amongst sub-Saharan African patients with ARNSHL.

There has been some contention on the role of *GJA1* in hearing loss with initial reports being subsequently discredited as the researchers had initially failed to distinguish between the gene from the pseudogene; no pathogenic mutations in *GJA1* coding region in Africans from Cameroon and South Africa [56].

#### Other genes involved in nonsyndromic hearing loss

Outside of connexin genes, there have been mutations reported in various other genes. Table 2 summarises a few of these in according to their role or function. Many of the mutations were found amongst Caucasian, Asian and Middle-Eastern populations [57]. To the best of our knowledge, there are neither data reported from sub-Saharan populations with ARNSHL nor any recurrent mutations in sub-Saharan populations.

**Table 2:** Genes implicated in cases of autosomal recessive non-syndromic hearing loss

Function	Genes
Cochlear Homeostasis	Gap Junctions: *GJB2, GJB6, GJB3*
	Tight Junctions: *CLDN14, TRIC*
	*SLC26A4*
Cellular Organization	Myosins: *MYO3A, MYO6, MYO7A, MYO15A*
	*TRIOBP, WHRN, USH1C, CDH23*
Tectorial Membranes Associated Proteins	*TECTA, COL11A2, STRC*
Neural transmission	*OTOF, PJVK*
Other or Unknown function	*TMC1, TMPRSS3, LOXHD1, PDZD7, GIPC3*

Adapted from [57]

Due to the number of different genes and mutations that have been implicated in hearing loss, it has become imperative that methods that are able to interrogate many gene positions at the same time be employed to find the relevant variants in African populations. Thus, the obvious choice becomes sequencing, especially using next-generation sequencing (NGS).

### Exploration of hearing loss genes using Next-Generation sequencing

#### Whole exome sequencing

Massive parallel sequencing allows for the interrogation of multiple genes at the same time. This can be whole genome sequencing where by the entire 3 billion base pairs of the individual's genome are sequenced. With most monogenic disorders having been caused by exonic mutations, whole exome sequencing is often favoured. Moreover, exonic regions represent only 1% of the entire genome which makes this interrogation both time and financially feasible as compared to whole genome sequencing [58]. This involves selecting only the exons of the genome to sequence and screen for mutations or variations. This approach was recommended for autosomal recessive monogenic disorders’ mutation screening [59].

The use of consanguineous families also helps narrow the search for such mutations as one has to look for homozygosity and the close relatedness of the family members’ helps in distinguishing between variations and disease related mutations. Other approaches involve using unrelated individuals whom present with the same clinical symptoms and comparing their captured variants to decide whether or not they are significant [58].

The use of gene mapping has long been a standard principle to try and identify the gene associated with a disease. In 2010 Walsh Et al. used homozygosity mapping and whole exome sequencing to identify the causative gene on locus DFNB82 in a Palestinian family with nonsyndromic hearing loss [60]. The region of interest contained 5 genes and spun 3.1Mb on chromosome 1p13.1. The design used to capture data from exonic regions covered 38Mb of the human gene which encompassed 23, 739 genes. Two of the 5 genes could not be evaluated as they lay on segmental duplications. 80 variants which passed the quality threshold were reported on the remaining three genes. Only 7 had not been previously reported on thedbSNP database. Ultimately p.R127X of GPSM2 was identified as the causative mutation on this locus [60].

In the Middle East, 30 individuals from 20 consanguineous families were selected for whole-exome sequencing analysis [61]. The families were from Iran and Turkey. On average each sample yielded more than 90 000 point mutations from whole-exome sequencing before any filters were applied [61]. There after only 12 homozygous mutations from known deafness genes were reported in 12 families [61]. They also report 4 novel heterozygous mutations in the 12 families where homozygous mutations had already been reported. They recommend the exploration of heterozygous mutations within known implicated genes when seeking causative mutations in small families or sample sizes [61].

The drawback of using Whole exome sequencing is that you select for the entire exome which will also carry genes not related to your disease of interest. One might also enrich for areas of less importance while losing genes which could be of interest or importance. The filtering of variants leaves a few variants to be interrogated. There is also the risk of losing rare variants. If one does not have family members’ or parents’ DNA to also work with, the efficiency is also negatively affected. A better approach would be to select for genes or specific areas of interest to screen rather than the entire exome, especially if associations for the disease have already been made.

#### Targeted gene sequencing

When the genes associated with a particular disorder have already been identified, targeted exome sequencing could be the most appropriate subsequent step in clinical settings. This involves using DNA chips and arrays with the desired genes already selected to capture DNA from those selected regions [58]. This fine tunes the approach as only specific genes are interrogated. This setting also provides a way for diagnostic tools to be developed to be used in clinical settings [62].

Genes associated with ARNSHL have been identified in many populations around the world [57]. These results further demonstrated the heterogeneity of ARNSHL thus using a targeted increases the likelihood of finding causative mutations amongst other populations. A tool has been developed which targets genes known to be associated in ARNSHL for exome sequencing [62]. The tool is named OTOScope and is reported to have the sensitivity and feasibility to rapidly diagnose and resolve cases of ARNSHL [62]. It is a DNA Microarray Chip which enriches exonic data of more 67 genes which have been implicated in nonsyndromic hearing loss and in Usher syndrome [62]. Massive parallel sequencing of these regions allows for the resolution of ARNSHL and Usher Syndrome. It has also shown to be sensitive and specific enough for use in clinical settings.

The developers of the tool demonstrated the sensitivity and specificity of their tool by screening three positive controls and one negative control [62]. Upon validation of their results (by Sanger sequencing) to the known genotypes of the controls, they screened 6 unknowns i.e. unresolved cases [62]. Pathogenic mutations were identified in 5 of the unknowns, three of which had not been previously reported [62]. Subsequently, 100 new samples with an apparent genetic deafness were screened using OTOScope. An overall diagnostic rate of 42% was reported for this second study [63].

Another next generation sequencing tool has been developed called OtoSeq [64]. It targets about 24 genes associated with sensorineural hearing loss. The targeted genes are enriched using microdroplet polymerase chain reaction [65]. It was used to resolve hearing loss in a sub-set of a large Pakistani cohort of 243 families. Thirty four families were selected for the screening using OtoSeq based on co-segregating with markers for *MYO7A, SLC26A4* and *CDH23*. Twenty fourmutations were identified in 28 families. Eleven of these mutations were novel *MYO7A* mutations [66].

This method of targeted enrichment and massively parallel sequencing has been adopted by other research groups to resolve cases hearing loss. Amongst them investigators of the Chinese population, Yang et al, have demonstrated the success of using targeted exome sequencing. Their technique used exonic data from 79 deafness genes [67]. The 125probands used were excluded of mutations in the most common deafness genes amongst this population namely *GJB2, SLC26A* and a mitochondrial mutation in *MT-RNR1*. They report 45 novel recessive mutations and three novel dominant mutations. They also put emphasis on the fact that17.4% of these novel mutations were found in less commonly screened genes advocating for more genetic testing to be done on these genes [67]. Amongst the Japanese, 216 patients with bilateral sensorineural hearing loss were recruited. One hundred and twelve genes were selected for targeted genesequencing. They report 57 genes to have been identified as responsible for the loss of hearing in their cohort [68]. Amongst the top candidate genes with the highest number of mutations were *GJB2, SLC26A4, USH2A, GPR98, MYO15A, COL4A5 and CDH23* [68]. Eighty six point six percent (86.6%) of the patients carried at least one mutation and 69 patients’ hearing loss was resolved [68]. More than 250 mutations were confirmed by Sanger sequencing.

## Conclusion

These are a few examples of the use of NGS to uncover mutations responsible for cases of hearing loss across various population groups. Upon the identification of such mutations, there can be a step towards population specific diagnostic tool development. There is very little data available on the genetic profile of hearing loss patients in Sub-Saharan Africa; with the current investigatory route being NGS in resolving this matter, it is the only way forward.
